# Economic evaluation of a lifestyle intervention in primary care to prevent type 2 diabetes mellitus and cardiovascular diseases: a randomized controlled trial

**DOI:** 10.1186/1471-2296-14-45

**Published:** 2013-04-04

**Authors:** Marieke F van Wier, Jeroen Lakerveld, Sandra D M Bot, Mai J M Chinapaw, Giel Nijpels, Maurits W van Tulder

**Affiliations:** 1 Department of Epidemiology and Biostatistics and the EMGO Institute for Health and Care Research, VU University Medical Center, Amsterdam, The Netherlands; 2 Departments of General Practice and Elderly Care and the EMGO Institute for Health and Care Research, VU University Medical Center, Amsterdam, The Netherlands; 3 Departments of Public and Occupational Health and the EMGO Institute for Health and Care Research, VU University Medical Center, Amsterdam, The Netherlands; 4 Department of Health Sciences and the EMGO Institute for Health and Care Research, VU University, Amsterdam, The Netherlands; 5 Faculty of Earth and Life Sciences, Department of Health Sciences, VU University, De Boelelaan 1085, 1081 HV, Amsterdam, The Netherlands

**Keywords:** Cost-effectiveness, Cost-utility, General practitioner, Lifestyle counseling, Practice nurse

## Abstract

**Background:**

Cost-effectiveness studies of lifestyle interventions in people at risk for lifestyle-related diseases, addressing ‘real-world’ implementation, are needed. This study examines the cost-effectiveness of a primary care intervention from a societal perspective, compared with provision of health brochures, alongside a randomized controlled trial.

**Methods:**

Adults aged 30-50 years, at risk of type 2 diabetes (T2DM) and/or cardiovascular disease (CVD), were recruited from twelve general practices in The Netherlands. They were randomized to the intervention (n = 314) or control group (n = 308). The intervention consisted of up to six face-to-face counseling sessions with a trained practice nurse, followed by three-monthly sessions by phone. Costs were collected using three-monthly retrospective questionnaires. Quality of life was measured with the EuroQol-5D-3L, at baseline, 6, 12 and 24 months. Nine-year risk of developing T2DM and ten-year risk of CVD mortality were estimated using the ARIC and SCORE formulae, respectively, based on measurements at baseline and 24 months while applying a fixed age of 60 years at both time points.

**Results:**

Small, statistically non-significant differences in effects were found between the intervention and control group with regard to risk scores and Quality Adjusted Life Years (QALYs) gained. The mean difference in costs between the intervention and control group was €-866 (95% confidence interval -2372; 370). The probability that the intervention was cost-effective varied from 93% at €8000/QALY to 88% at €80,000/QALY.

**Conclusion:**

A primary care lifestyle intervention aimed at adults at increased risk of T2DM and/or CVD could result in cost savings over a two-year period. However, due to methodological uncertainty no advice can be given regarding the implementation of the intervention in Dutch general practices.

**Trial registration:**

Current Controlled Trials ISRCTN59358434.

## Background

Worldwide an increased prevalence of type 2 diabetes (T2DM) has been noted over the last decades [1]. A further rise is expected, due to an aging population and the high prevalence of obesity. The expected increase is not only a medical, but also a socioeconomic problem. In most countries health care costs are rapidly rising, and the obesity epidemic plays an important role in this process [2]. Several studies have shown that the risk of developing T2DM and associated cardiovascular diseases (CVDs) reduces with weight loss and improved lifestyle behaviors [3]. A recent review found evidence for the cost-effectiveness of diabetes prevention interventions [4]. Nevertheless, as these interventions have been studied under strictly controlled conditions, there remains a need for cost-effectiveness studies of interventions addressing ‘real-world’ settings. Information about the tradeoff between costs and benefits of these interventions will help policy makers to decide whether it is efficient to implement and reimburse them.

In the Netherlands, a theory-based primary care intervention aimed at reducing the risk of T2DM and CVD was developed and tested in general practices using a randomized controlled trial (RCT) design [5]. The purpose of the current study is to assess the cost-utility and cost-effectiveness of the intervention in reducing the risk for developing T2DM and CVD, compared with the provision of health brochures. The primary outcome of the study is the number of Quality Adjusted Life Years (QALYs) gained. Secondary outcomes are changes in estimated risk of developing T2DM in the following 9 years and of CVD mortality in the following 10 years. These outcomes are assessed within the two year trial period.

## Methods

### Design of the study

An economic evaluation was conducted alongside a RCT, the Hoorn Prevention Study, carried out in the Netherlands from 2008 to 2010. The economic evaluation was performed from a societal perspective. Details of the study design, the recruitment results and the implementation of the intervention have been published elsewhere [5,6]. The study design and informed consent procedure were approved by the Medical Ethics Committee of the VU University Medical Center and all participants provided written informed consent.

### Study population and setting

**Figure 1:**
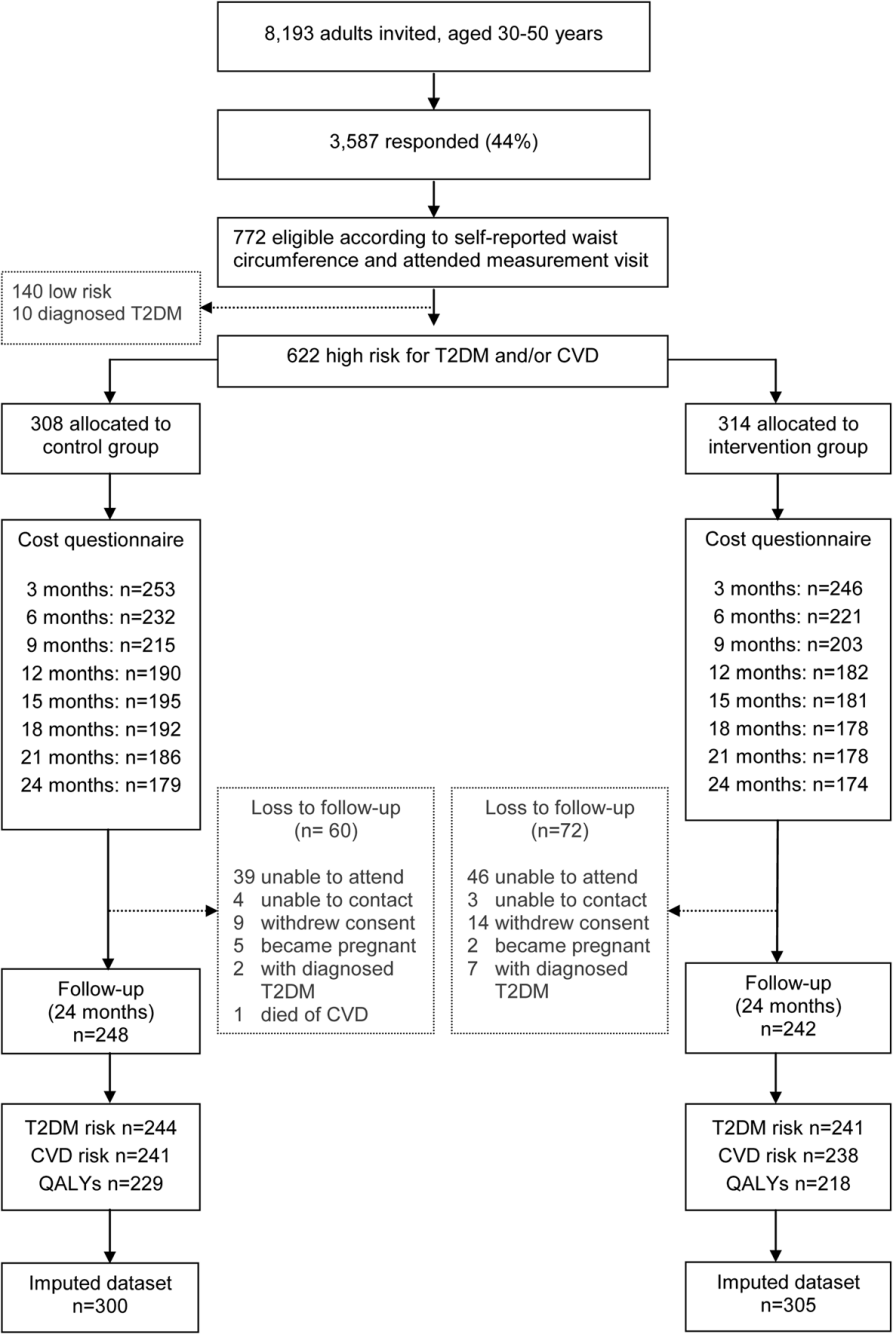
**Participant flow.** T2DM, Type 2 Diabetes Mellitus; CVD, Cardiovascular Disease, QALYs, Quality Adjusted Life Years. T2DM risk: the 9-year risk of developing T2DM; CVD risk: the 10-year risk of CVD mortality.

The study population consisted of men and women living in several municipalities in the semi-rural region around the city of Hoorn in the Netherlands. The region has over 200,000 inhabitants. Twelve general practitioners provided the addresses of 8193 of their patients, aged 30-50 without known diabetes or CVD, based on patient records. These persons were asked by mail to measure their waist circumference with a paper tape measure provided to them. Waist circumference reflects the magnitude of abdominal adipose tissue deposits as well as total fat mass [7]. It is considered to be a good measure with which to identify persons with an increased risk of developing T2DM and CVD [8]. Of the 3587 responders, 921 had an increased waist-circumference (≥101 cm for men and ≥87 cm for women). These 921 persons were invited for baseline measurements, of which 772 attended. At baseline, the at the age of 60 years anticipated 9-year risk of developing T2DM and the 10-year risk of CVD mortality were estimated, assuming all other risk factors would remain unchanged [5]. We did this by setting the age of all participants to 60 years in our risk score calculations. This projection was done to address the problem of a current low absolute risk due to a relative young age which, if no improvements occur, would develop to a high risk at age 60 [9]. Responders with a minimum estimated risk of 10% on one or both risk scores were eligible. Reasons for exclusion were pregnancy, unable to communicate adequately in the Dutch language or being diagnosed with T2DM or CVD. A total of 150 respondents were excluded, because of low risk (n = 140) or T2DM diagnosis (n = 10). A total of 622 participants were randomly assigned to either the intervention group (n = 314) or the control group (n = 308; Figure 1). Random allocation was done in blocks of 10 to keep the sizes of treatment groups similar, using a computerized random number generator. If there was more than one participant from the same family, the consecutive members of that family were allocated to the same group to avoid contamination. Randomization was performed by an independent administrative assistant from the Diabetes Research Center, who was not involved in the intervention, measurements or analyses.

### Intervention and control

Both groups received care as usual, e.g. prescription of medication in the case of severe hypertension. Additionally, an intervention based on cognitive behavioral principles was offered to the intervention group. The intervention was aimed at improving physical activity, diet or smoking behavior, as chosen by the participants. It consisted of up to six monthly individual 30-minute face-to-face counseling sessions, followed by three-monthly 15-minute sessions by phone. Counseling was done by eight practice nurses, who received 18 hours of training prior to the intervention. Feedback on the quality of counseling was provided to the practice nurses halfway through the sessions and consisted of one hour individual coaching. Also, a peer supervision meeting was arranged. A full description of the intervention and its underlying principles has been published elsewhere [5].

The control group received existing brochures containing health guidelines regarding physical activity and a healthy diet, obtained from the Dutch Heart Foundation. Smokers received an additional brochure on how to stop smoking from the Dutch expertise center for tobacco discouragement policies (STIVORO). An independent administrative assistant sent the brochures to the participants.

### Study measures

#### Clinical outcome measures

For the cost-utility analysis, the EuroQol-5D with three levels for each of the five health dimensions (EQ-5D-3L) was used to assess quality of life at baseline, and at 6, 12 and 24 month follow-up [10]. Health utilities were estimated with the Dutch tariff [11]. QALYs were calculated by multiplying the utilities with the amount of time a participant spent in a particular health state. Transitions between health states were linearly interpolated.

Outcome measures of the cost-effectiveness analyses were the estimated risk of developing T2DM and the estimated risk of CVD mortality. The 9-year risk of developing T2DM was estimated with the risk formula derived from the Atherosclerosis Risk In Communities (ARIC) Study, based on ethnicity, parental history of diabetes, systolic blood pressure, waist circumference, and height [12]. This formula was selected because of its potential applicability in primary care, since it includes only non-invasive methods to assess participants’ T2DM risk. The 10-year risk of CVD mortality was estimated with the formula developed by the Systematic COronary Risk Evaluation (SCORE) project [9] which includes sex, smoking status, total cholesterol, and systolic blood pressure. The application of the SCORE formula was considered to be the most useful for the calculation of CVD mortality risk. Former comparative analyses of the SCORE, the Framingham and the UKPDS formulae demonstrated that the Framingham function may overestimate an individual’s absolute chronic heart disease risk, and the UKPDS formula was not chosen based on its specific inclusion of T2DM as variable [13]. Baseline risks and follow-up risks were estimated while standardizing the age of each participant at 60 years. The formulas and the estimated risks are further explained in Additional file 1. Data was collected by means of physical measurements and questionnaires. A detailed description of the data collection has previously been published [5].

#### Cost measures

Information on health care utilization, medication costs, participant costs and productivity loss was obtained through eight retrospective three-month questionnaires provided to the participants between baseline and 24-month follow-up.

Health care utilization consisted of general practitioner care, allied health care, medical specialist care and hospitalization [14]. Participants’ costs concerned complementary care, over-the-counter (OTC) medication, and costs associated with improving physical activity, such as sports club memberships and sports equipment. Health care utilization and complementary care were valued with Dutch standard costs. When these were not available, prices reported by professional associations were used. The costs of prescribed medication were calculated using prices charged by the Royal Dutch Society for Pharmacy [15]. Costs of OTC medication and sports were reported by the participants. Costs of productivity losses based on self-reported sick leave from work were estimated with the friction cost approach (friction period 154 calendar days and an elasticity of 0.8), using the mean income of the Dutch population according to age and gender [14]. Cost categories and prices used in the economic evaluation can be found in Additional file 2. Prices were adjusted to the year 2008 (the year of the first measurement) using consumer price indices [16]. In the base case analysis no discounting was done for the costs in the second year. The effect of discounting was however explored in a sensitivity analysis.

#### Intervention costs

Bottom-up micro-costing was used to estimate intervention costs. These consisted of a sum of fixed costs and variable costs. The fixed costs covered costs of the development and printing of materials, training and supervision of the practice nurses, and of costs for selecting and inviting the participants. Of the 207,000 inhabitants in the region, around 27% is between 30 and 50 years old. Of these 56,000 people, about one-fifth is expected to be eligible and willing to participate. Development costs were therefore spread over 10,000 patients. Total fixed costs per participant were €15. Variable costs per participant consisted of counseling costs. These were calculated based on the number of face-to-face contacts and telephone contacts that took place as reported by the practice nurse. The cost price of a face-to-face session was €24, and of a phone session €12.

### Statistical analyses

#### Multiple imputation

Analyses were based on group allocation, regardless of actual intervention received or of adherence to the intervention (i.e. intention-to-treat). In the base case analysis it was assumed that when data on follow-up telephone sessions were missing, no sessions had taken place and the missing data were imputed with zero. The reason for doing so was that many practice nurses failed to report the number of follow-up phone contacts they had with a particular participant. It seems fair to assume that this was because none took place. Missing number of face-to-face contacts, health care costs, participant costs, sick leave days and clinical outcomes were imputed using multiple imputation techniques [17]. The imputation model included age, sex, educational level, smoking status, living alone yes/no, baseline outcome values, available midpoint (6 and 12 months) and follow-up outcome values, and available number of face-to-face and phone sessions, health care costs, participant costs and sick leave days at each measurement. Imputations were done separately for the intervention and control group. Five different data sets were created in SPSS (version 17.0.2, Chicago, Ill) using Fully Conditional Specification and Predictive Mean Matching procedures, assuming that data were missing at random [18]. These data sets were analyzed as specified below. The estimates were pooled with methods described by Rubin [19]. This method does not allow for an estimation of standard deviations, so the standard error of the mean (SEM) is presented to describe variability.

#### Base case analysis

Costs and outcomes were estimated separately. Linear regression analysis (ordinary least squares) was used to compare outcomes between the intervention and control groups. Follow-up outcomes were adjusted for baseline values [20]. To compare costs between groups, confidence intervals around the mean differences in costs were estimated using the bias-corrected and accelerated bootstrap method with 5000 replications. Incremental cost-effectiveness ratios (ICERs) were estimated by dividing the difference in total costs over 24 months between the treatment groups by the regression coefficient for treatment effect at 24 months. To graphically present uncertainty around the ratios, bias-corrected percentile bootstrapped cost-effect pairs (5000 replications) were plotted in cost-effectiveness planes (CE-planes) [21]. The uncertainty of cost-effectiveness was presented in cost-effectiveness acceptability curves (CEACs) [22]. Analyses were performed with R version 2.10.1 [23]. Participants who developed CVD or T2DM during the study (n = 9) or became pregnant (n = 7), and participants who had died (n = 1) were excluded from all analyses.

#### Sensitivity analyses

Sensitivity analyses were conducted to test the robustness of the results. In the first sensitivity analysis the costs for the second year were discounted with 4% and QALYs achieved in this year were discounted with 1.5%, in line with Dutch guidelines [24]. In the second sensitivity analysis the productivity losses were valued with the human capital method. In the third sensitivity analysis the costs for sports were excluded. In the fourth sensitivity analysis, missing data for follow-up phone calls (which in the base case analysis were assumed to be zero) were multiply imputed. For the fifth sensitivity analysis, 50 multiply imputed files were created, according to the rule of thumb that the number of imputed files should at least equal the fraction of incomplete cases [25]. The sixth sensitivity analysis was restricted to participants with complete cost and effect data, i.e., complete case analysis (CCA). In the final sensitivity analysis, cost-effectiveness was assessed from the healthcare perspective.

## Results

### Participant flow and baseline characteristics

The participant flow is presented in Figure 1. On average, 64% of the cost questionnaires were fully completed and returned. Data on the number of face-to-face and phone counseling sessions, necessary to calculate the intervention costs, were complete for, respectively, 291/314 (93%) and 177/314 (56%) participants. As a consequence, 36 to 44% of the cost data were imputed, as was 22 to 26% of the outcome data.

Baseline characteristics are given in Table 1.

**Table 1:** Baseline characteristics of all randomized participants

	**Control group**	**Intervention group**
	**n = 308**	**n = 314**
Female [n (%)]	185 (60.1)	178 (56.7)
Age [mean (SD), (years)]	43.4 (5.5)	43.6 (5.1)
Level of education [n(%)]^a^		
≤ Primary education	103 (33.6)	101 (32.5)
Secondary education	145 (47.1)	141 (44.9)
College, university	59 (19.2)	69 (22.0)
Paid job [n (%) yes]^b^	269 (87.9)	262 (85.6)
Smoking [n (%) yes]^a^	54 (17.5)	74 (23.9)
T2DM risk [mean (SD)], %	18.8 (8.5)	19.0 (7.8)
CVD risk [mean (SD)]^e^, %	3.8 (2.9)	4.0 (3.0)
Health utility [mean (SD)]^f^	0.90 (0.13)	0.88 (0.16)

*T2DM*, Type 2 Diabetes Mellitus; *CVD*, Cardiovascular Disease. ^a^ n = 617; ^b^ n = 612; ^c^ The at the age of 60 years anticipated of developing T2DM in the following 9 years; ^d^ The at the age of 60 years anticipated risk of CVD mortality in the following ten years; ^e^ n = 619; ^f^ n = 612,

### Intervention compliance

The mean number of face-to-face counseling sessions after multiple imputation was 2.4 (SEM 0.08). The mean number of follow-up phone sessions was 2.3 (SEM 0.2). The findings of our process evaluation indicate that practice nurses adhered to the intervention protocol and were reasonably competent and confident in the provision of the intervention [6].

### Clinical outcomes

QALYs achieved during the two-year period were 1.81 (SEM 0.01) in the control group, and 1.80 (SEM 0.02) in the intervention group. After adjustment for baseline utilities, the intervention group gained a statistically nonsignificant 0.02 (95% CI -0.02 to 0.05) more QALYs than the control group.

Both groups improved their risk of T2DM and CVD. Respectively, these reduced to 17.9% (SEM 0.5) and 3.6% (SEM 0.2) in the control group, and 18.5% (SEM 0.5) and 3.7% (SEM 0.2) in the intervention group. Only minimal and statistically non-significant differences were found between the groups. The intervention group experienced a relative increase in T2DM risk of 0.6% (95% CI -0.1 to 1.3) and a decrease in CVD risk of 0.1% (95% CI -0.4 to 0.2), compared with the control group.

### Costs

The mean costs and cost differences in each cost category are presented in Table 2. Intervention costs were €98 (SEM 2.4) per participant. The majority of participant costs consisted of costs for sports and sports equipment (88%). Indirect costs of productivity losses ranged from €0 to €71,695 in the control group and €0 to €45,195 in the intervention group. Statistically significant differences in costs between the groups were not seen. The direction of the cost-differences in each category was consistently in favor of the intervention group. The overall cost-difference was €-866 (95% CI -2392 to 370).

**Table 2:** **Pooled costs and cost differences in Euros**^**a **^**between baseline and two year follow-up, after multiple imputation**

	**Control group**	**Intervention group**	**Intervention versus control**
	**N = 300**	**N = 305**	
	**Mean (SEM)**	**Mean (SEM)**	**Mean difference (95% CI)**
Healthcare costs	1021 (107)	1016 (92)	−5 (-316;272)
*Intervention*	*0*	*98 (2.4)*	*98 (NA)*
*Other*	*1021 (107)*	*918 (92)*	*−104 (-414;173)*
Participant costs	774 (69)	642 (45)	−132 (-323;27)
Productivity losses	2918 (528)	2189 (340)	−729 (-2008;285)
Total	4713 (626)	3847 (388)	−866 (-2400;370)

*NA*, Not Applicable; *SEM*, Standard Error of the Mean.

^a^ All costs are given in Euros adjusted to the year 2008.

### Cost-utility

**Figure 2:**
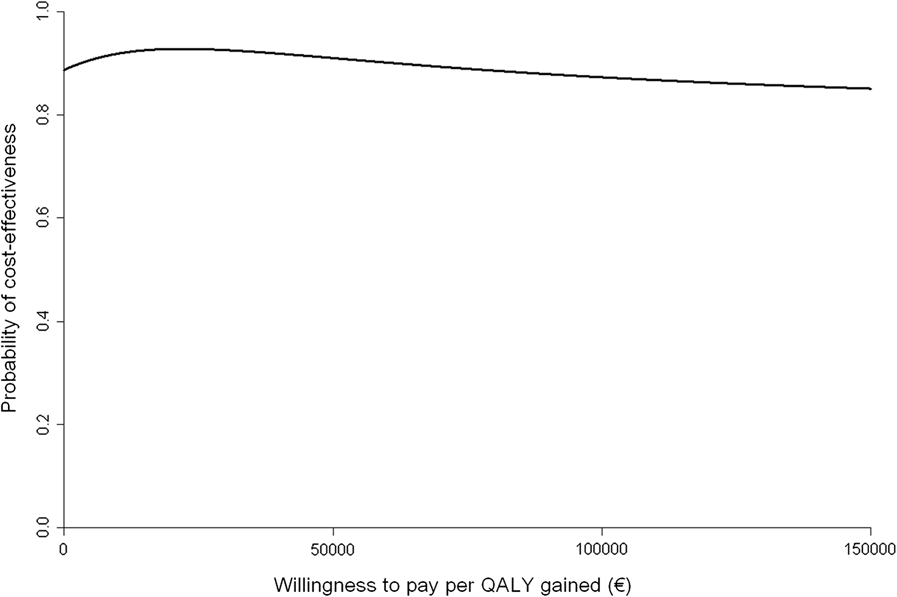
Cost-effectiveness acceptability curve for Quality Adjusted LifeYears (QALYs) gained.

The incremental cost-effectiveness ratio of €-50,273 per QALY gained was reflective of a small gain in QALYs (0.02) in the intervention group, at a reduction in societal costs (€-866), as compared with the group who received health brochures (Table 3). Almost 77% of cost-effectiveness pairs were in the south-east quadrant, indicating likeliness for greater effectiveness at lower costs. The CEAC showed the probability that the intervention is cost-effective compared with health brochures was 89% at a ceiling ratio of €0 per additional QALY gained, 93% at €8000/QALY and 89% at €80,000/QALY (Figure 2). This rise and subsequent fall in the probability of cost-effectiveness can be explained by looking at the distribution of the cost and effect pairs in the CE-plane. As the willingness to pay increases, the joint density in the north-east quadrant (8.2%) is included as cost-effectiveness before the joint density in the south-west quadrant (12.9%) is excluded as no longer cost-effective and the probability of cost-effectiveness drops [26].

**Table 3:** Incremental cost-effectiveness ratios and distribution of the joint cost-effect pairs in the cost-effectiveness plane

	**Analysis**^**a**^	**Sample size per group**		**ΔC (95% CI)**	**ΔE (95% CI)**		**Distribution in cost-effectiveness plane (%)**			
		**Control**	**Intervention**	**Euros**		**ICER**	**NE**^**b**^	**SE**^**c**^	**SW**^**d**^	**NW**^**e**^
**T2DM risk**^**f**^	Base case	300	305	−866 (-2372;370)	0.6 (-0.1;1.3)	-1416	0.6	4.1	85.9	9.4
	Complete cases	117	105	−30 (-2171;1446)	0.7 (-0.4;1.7)	-44	5.1	4.4	45.0	45.5
	Health care perspective	300	305	−5 (-316;272)	0.6 (-0.1;1.3)	−8	2.2	2.4	47.5	47.9
**CVD risk**^**g**^	Base case	300	305	−866 (-2372;370)	−0.1 (-0.4;0.2)	6405	8.0	74.3	15.4	2.3
	Complete cases	116	104	−19 (-2253;1410)	−0.03 (-0.34;0.29)	642	29.5	27.8	21.3	21.5
	Health care perspective	300	305	−5 (-316;272)	−0.1 (-0.4;0.2)	38	40.1	42.4	8.0	9.5
**QALY**	Base case	300	305	−866 (-2372;370)	0.02 (-0.02;0.05)	-50,273	8.2	76.8	12.9	2.1
	Complete cases	114	98	110 (-2004;1611)	0.02 (-0.02;0.06)	4770	46.4	40.6	4.2	8.7
	Health care perspective	300	305	−5 (-316;272)	0.02 (-0.02;0.05)	−298	40.7	44.7	5.0	9.6

ΔC = mean difference in total costs between the intervention group and control group in Euros adjusted to the year 2008; ΔE = mean difference in outcome; ICER is calculated as ΔC/ΔE. *ICER*, Incremental Cost-Effectiveness Ratio; *NE*, north-east; *SE*, south-east; *SW*, south-west; *NW*, north-west; T2DM, Type 2 Diabetes Mellitus; *CVD*, Cardiovascular Disease; *QALY*, Quality Adjusted Life Years.

^a^ The base case analysis and complete case analysis are based on the societal perspective. In the base case analysis and the analysis from the health care perspective missing data were multiply imputed. The complete cases analysis was restricted to participants with complete data on costs and the particular clinical outcome. ^b^ NE quadrant: the intervention is more effective and more costly than usual care. ^c^ SE quadrant: the intervention is more effective and less costly than usual care. ^d^ SW quadrant: the intervention is less effective and less costly than usual care. ^e^ NW quadrant: the intervention is less effective and more costly than usual care. ^f^ The at the age of 60 anticipated risk for developing T2DM in the following 9 years . ^g^ The at the age of sixty anticipated risk of CVD mortality in the following 10 years.

### Cost-effectiveness

The ICER of the 9-year risk for developing T2DM was -1416. Over the two year period, a €1416 saving was seen per 1% increase in risk for developing T2DM among the intervention participants as compared to those who received health brochures. The CE-plane showed that the majority (86%) of the cost-effectiveness pairs were located in the south-west quadrant, indicating that the intervention is likely to be less expensive, but also less effective than the alternative (Table 3). The CEAC for T2DM-risk (not presented) showed that the probability of cost-effectiveness at a ceiling ratio of €0 per one percent risk modification was 90%. The CEAC dropped when the amount that society is willing to pay or accept increased. Beyond a ceiling ratio of approximately €1500 per one percent risk change, the probability that health brochures are cost-effective is higher than the probability that the lifestyle intervention is cost-effective.

The ICER for the 10-risk of CVD mortality was 6405, meaning that a 1% lower risk as a result of the intervention is accompanied by a societal saving of €6405, compared with health brochures (Table 3). Cost-effectiveness pairs were mostly located (74%) in the south-east quadrant, indicative of higher effectiveness at lower cost (Table 3). The probability of cost-effectiveness for CVD-risk started at 90% at a ceiling ratio of €0 per one percent risk modification and fell slightly to 80% at a ceiling ratio of €10,000 per one percent risk modification (CEAC not presented).

### Sensitivity analyses

The sensitivity analyses in which costs and QALYs were discounted, productivity losses were valued with the human capital method, the costs for sports were excluded from the participant costs, missing number of follow-up phone calls were multiply imputed, and 50 multiply imputed data sets were created and analyzed, showed similar results (Results shown in Additional file 3). The sensitivity analysis in which only participants with complete cost and effect data were included and the analysis from the healthcare perspective, gave different results (Table 3). In the complete case analysis the difference in societal costs virtually disappeared, but the differences in the clinical outcomes were similar to the main analysis. The CEAC showed that for risk of T2DM that the probability of cost-effectiveness at a ceiling ratio of €0 was 50%. The CEAC for risk of CVD was around 50% at all ceiling ratios. With regard to QALYs gained, the maximum probability of cost-utility compared with health brochures was 45% at a ceiling ratio of €0/QALY, 75% at €80,000/QALY and converged to 77% at €100,000/QALY.

In the analysis from the healthcare perspective, the cost difference between the groups reduced to €-5. For all outcomes, the probability of cost-effectiveness was around 50% at a ceiling ratio of €0. For T2DM the probability decreased as willingness to pay increased, whereas for CVD it increased to nearly 70% at €10,000 per 1% change. Probability of cost-utility was about 80% from €25,000/QALY upwards, converging to 85% at the maximum willingness to pay.

## Discussion

This study examined the cost-effectiveness of a primary care intervention to prevent T2DM and CVD from a societal perspective, compared with provision of health brochures. Small, statistically non-significant differences in risk scores and QALYs gained were found between the intervention and comparison group. The mean difference in costs was €-866 (95% CI -2372 to 370). The probability of cost-effectiveness for health risk modification was 90% at a ceiling ratio of €0. Increasing the ceiling ratio resulted in a slight drop in the probability of cost-effectiveness for risk of CVD, but severely for risk of T2DM. The probability of cost-utility of the intervention, compared with the brochures group, was around 90% at all ceiling ratios. The results were confirmed in the sensitivity analyses, except in the complete case analysis. This showed that the intervention was not more likely to be cost-effective for reducing health risks than provision with health brochures. Furthermore, the probability of cost-utility reduced to a maximum of 77%.

### Clinical outcomes

The results indicate that there were no statistically significant differences between the intervention and control group with regard to the clinical outcomes. Baseline health utilities were already high, which implied that there was little room for improvement in QALYs gained. High baseline utilities and lack of improvement might be related to the use of the EQ-5D-3L to measure health status. This instrument is known for its ceiling effects in relatively healthy populations. Other instruments, such as the recently developed EQ-5D-5L might resolve this problem, but this is still under research [27]. Lack of differential effect on risk profile could be related to the low attendance to the counseling sessions. On average only 2.4 of the 6 available face-to-face counseling sessions had taken place, whereas the mean use of the 3-monthly phone-calls was 2.3. These results underscore the difficulties in translating efficacious methods to interventions that are feasible and effective in real-world settings.

### Costs

Cost-differences were in favor of the intervention albeit statistically not significant. Due to the skewed nature of the cost data, the study may have been underpowered to reach statistical significance for cost differences. The difference in total costs was mostly explained by differences in costs of productivity losses. To explore the effect of outliers on these costs, a post-hoc analysis was done. In this, the number of sick leave days was truncated at 30 days, as it is improbable that sick leave over 30 days would have been influenced by the intervention. The cost difference reduced to €-179 (95% CI -725 to 311). However, a reduction in societal costs remained.

To our knowledge, no other studies of lifestyle interventions to prevent T2DM or CVD have found immediate cost reductions as a consequence of the program, in the absence of health effects [4]. Some researchers have suggested that health promotion programs have non-health benefits that are currently not measured, such as increased health literacy [28]. These benefits may have a direct influence on health care use and sickness absenteeism [29]. The reduction in costs of personal expenses, mainly consisting of sports costs, is puzzling. Because the intervention participants were stimulated to be more physically active, higher costs were expected. All in all, the finding of possible cost savings in favor of the intervention cannot be easily explained.

### Cost-utility

The main aim of an economic evaluation is to decide whether the treatment under scrutiny offers value for money. Ceiling ratios for reductions in risk of CVD or T2DM are not determined. The ceiling ratio for QALYs gained in the Netherlands is also not established, but it has been proposed to be set at €8000 for diseases with a low burden and at €80,000 for diseases with a high burden [30]. The immediate burden of disease of an elevated risk for T2DM and CVD is unknown, but likely lies at the lower end of the range. At a ceiling ratio of €8,000 per QALY gained, the probability of cost-utility of the intervention was 93%. At the higher end, probability of cost-utility was 89% at a ceiling ratio of €80,000 per QALY gained. The intervention therefore has a high probability of cost-utility at all acceptable ceiling ratios. However, there is methodological uncertainty regarding this probability. The complete case analyses showed that the probability that the true cost-utility ratio falls below €80,000 is 75%. The analysis from the health care perspective also showed a lower probability of cost-effectiveness. The post-hoc analysis in which the number of sick leave days was truncated at 30 days, showed a maximum probability of 70% at a ceiling ratio of €0. Thus, in these sensitivity analyses, cost-utility results were less positive. Furthermore, people generally demand larger compensations for losses compared with how much they are willing to pay for gains (willingness to pay < willingness to accept). If this aversion to loss is applicable to losses in health, a lower probability of cost-utility would have been found [31]. Finally, as explained before, questions remain about the cause of the cost-differences. In light of these uncertainties, it is unsure if the possible benefits outweigh the efforts involved in the implementation of this new intervention.

### Limitations and strengths

A limitation of the study is the amount of incomplete cost data. Intervention costs were missing for 140/314 (45%) participants, mainly because the practice nurses failed to report the number of phone sessions to the research team. However, a more complete report may be difficult to achieve in real-world settings. In addition, 36% of the self-reported data on health care utilization, personal spending and sick leave was missing. This is comparable to other RCTs in the Netherlands with a follow-up of one year or longer [32,33]. Studies in the Netherlands that need individual data on health care use have to rely on self-report because it is not feasible to collect these data from health insurers. Nevertheless, methods to improve data-completeness in studies with many self-reported cost-measurements should be devised to increase the internal validity of these studies.

Over one-third of all cost data were imputed, using multiple imputation techniques. In multiple imputation it is assumed that unobserved data are (in part) dependant on the observed data (e.g. available costs). However, this assumption cannot be fully tested. Although methodological studies show that multiple imputation is preferred over complete case analyses and simple imputation methods results from this study should be treated with some caution.

The use of ‘projected’ risk scores for T2DM and CVD was useful to accentuate absolute risk, but should not be considered as current absolute risk. For both risk scores and for each participant, age was extrapolated to 60 years while all other variables in the risk scores (e.g. blood pressure or cholesterol) were absolute scores. Thus, the scores estimated the risk of participants as if they were 60 years old, but with current values.

Lastly, the time horizon of the study was too short to observe the development of T2DM or CVDs, and to measure their associated changes in QALYs achieved. Decision modeling could be used to extend the time horizon of the study. This would give more insight in the longer term cost-effectiveness, and would increase the comparability of the results to those from other studies. Decision modeling also has the advantage that the results of similar interventions could be included, thereby broadening the evidence base [34]. This would however have to be done in a separate study.

Strengths of the study include the relatively long-term follow-up in terms of intermediate outcomes, the use of multiple imputation to address the large amount of missing cost data, the use of both the friction cost approach and the human capital approach to value productivity losses, and the application of a randomized controlled design in a real-world setting.

## Conclusion

The lifestyle program offered by practice nurses to adults at risk for T2DM and CVD was not more effective in reducing these risks than the provision of general health brochures. However, the intervention appeared to result in short-term cost savings. A high probability of cost-utility was found at all ceiling ratios. Nevertheless, due to methodological uncertainty no advice can be given regarding the implementation of the intervention in Dutch general practices.

## Competing interests

The authors declare that they have no competing interests.

## Authors’ contributions

GN, SB and MvT designed the study, with help from MC. JL further worked out the study protocol and carried out the data collection. MvW did the data analysis and wrote the first draft. Interpretation of data and critical revision of the article were done by all co-authors, and all gave approval of the final version.

## Pre-publication history

The pre-publication history for this paper can be accessed here:

http://www.biomedcentral.com/1471-2296/14/45/prepub

## Supplementary Material

Additional file 1 **Calculation of SCORE and ARIC at baseline and 2 year follow-up.** In this file the SCORE and ARIC formulae are given, as well as the baseline and follow-up values of their underlying parameters. Click here for file

Additional file 2 **Price weights used for valuation of resource use, per visit unless otherwise mentioned. **This file contains the price weights that were used to value resource use. Click here for file

Additional file 3 **Incremental cost-effectiveness ratios and distribution of the joint cost-effect pairs in the cost-effectiveness plane of all analyses.** This file shows the results from the base case analysis and all sensitivity analyses. Click here for file
