# Supplementary material for: Economic evaluation of a lifestyle intervention in primary care to prevent type 2 diabetes mellitus and cardiovascular diseases: a randomized controlled trial
Source: BMC Fam Pract. 2013 Apr 4;14:45. doi: 10.1186/1471-2296-14-45 (PMC3662579; doi:10.1186/1471-2296-14-45)
Supplement: Additional file 1 — Calculation of SCORE and ARIC at baseline and 2 year follow-up. In this file the SCORE and ARIC formulae are given, as well as the baseline and follow-up values of their underlying parameters. [file 1471-2296-14-45-S1.pdf]

Additional file 1 – Calculation of SCORE and ARIC at baseline and 2 year follow-up

Calculating 10-year risk estimates for fatal CVD (SCORE)

Step 1

Calculate the underlying risks for coronary heart disease and for non-coronary cardiovascular disease separately for the person's age now (for this study, age now is set as 60) and for their age in ten years time, using the values for alpha and p shown in Table 1. The underlying survival probability, S0, is given by:

$S_0(60) = \exp\{-(\exp(\alpha))(60 - 20)^p\}$

$S_0(70) = \exp\{-(\exp(\alpha))(60 - 10)^p\}$

Step 2

Using the coefficients in Table 2, calculate the weighted sum, *w*, of the risk factors cholesterol, smoking and systolic blood pressure. Two weighted sums will have to be calculated, one for coronary heart disease and one for non-coronary cardiovascular disease. Smoking is coded as 1 for current and 0 for non-smoker, so no value for smoking has to be entered if the person is a non-smoker. Cholesterol is measured in mmol/L and SBP is measured in mmHg. The weighting for each risk factor is denoted by beta.

$w = \beta_{chol}(cholesterol - 6) + \beta_{SBP}(SBP - 120) + \beta_{smoker}(current)$

Step 3

Combine the underlying risks for coronary heart disease and for non-coronary cardiovascular disease, at the person's age and at their age ten years from now (four calculations) which were calculated in step 1 with the weighted sum of a person's risk factors from step 2 for the two end-points, coronary heart disease and non-coronary cardiovascular disease to get the probability of survival at each age for each cause.

$S(60) = \{S_0(60)\}^{\exp(w)}$

$S(70) = \{S_0(70)\}^{\exp(w)}$

Step 4

For each cause, calculate the 10-year survival probability based on the survival probability for the person's current age and their age in 10 years time:

$S_{10}(60) = S(70) / S(60)$

Step 5

Calculate the 10 year risk for each end-point as

$Risk_{10} = 1 - S_{10}(60)$

Step 6

Combine the risks for coronary heart disease and non-coronary cardiovascular disease by adding them:

$CVDRisk10(60) = [CHDRisk(60)] + [Non\text{-}CHDRisk(60)]$

	**Baseline (n=622)**		**2 years follow-up (n=585)**		**Δ baseline- follow-up (n=585)**		***P* between group**[a]
	C	I	C	I	C	I	
SCORE	3.8 (2.9)	4.0 (3.0)	3.5 (2.8)	3.8 (3.1)	-0.2 (1.4)*	-0.3 (1.8)*	ns
Sex (% female)	60.1	56.7	NA	NA	NA	NA	
Smoking (%)	17.6	17.2	14.9	16.2	14.9*	16.2*	ns
Total cholesterol	5.5 (1.0)	5.6 (1.0)	5.5 (0.95)	5.6 (1.0)	-0.1 (0.6)*	0.0 (0.7)	ns
Systolic blood pressure	129.3 (13.3)	128.7 (13.2)	126.5 (13.1)	126.9 (13.1)	-2.4 (11.6)*	-2.3 (11.3)*	ns

[a] *P*<0.05, adjusted for baseline; C = Control group; I = Intervention group; SCORE = Systematic COronary Risk Evaluation; NA = Not Applicable; ns = not significant; * = within group difference *P*<0.05

Calculating 9-year risk estimates of developing diabetes (ARIC)

The following are parameter estimates for the models estimating the probability of developing diabetes over a 9-year follow-up period:

Pr(DM) = 1/(1 + e^{-x}), where x = –7.3359 + 0.0271 x 60 (fixed age) + 0.2295 x black + 0.5463 x parental history of diabetes + 0.0161 x systolic blood pressure (mmHg) + 0.0412 x waist (cm) – 0.0115 x height (cm).

Black = 1 if Negroid, 0 if white, and parental history of diabetes = 1 if at least one parent has diabetes or 0 if not.

N.B. All participants in this study were white.

	**Baseline (n=622)**		**2 years follow-up (n=585)**		**Δ baseline - follow-up (n=585)**		***P* between group[a]**
	C	I	C	I	C	I	
ARIC	18.8 (8.5)	19.0 (7.8)	17.5 (7.9)	18.5 (7.7)	-0.9 (4.4)*	-0.4 (3.9)	ns
Parental history of DM (%)	25.0	29.9	NA	NA	NA	NA	
Systolic blood pressure	129.3 (13.3)	128.7 (13.2)	126.5 (13.1)	126.9 (13.1)	-2.4 (11.6)*	-2.3 (11.3)*	ns
Waist circumference	96.7 (9.7)	96.7 (9.8)	95.6 (9.6)	96.4 (10.2)	-0.7 (5.0)*	0.0 (5.0)	ns
Height (cm)	176.3 (10.0)	176.6 (10.3)	NA	NA	NA	NA	

[a] *P*<0.05, adjusted for baseline; C = Control group; I = Intervention group; ARIC = Atherosclerosis Risk In Communities; NA = Not Applicable; DM = Diabetes Mellitus; ns = not significant; * = within group difference *P*<0.05
