# Supplementary material for: Economic evaluation of a lifestyle intervention in primary care to prevent type 2 diabetes mellitus and cardiovascular diseases: a randomized controlled trial
Source: BMC Fam Pract. 2013 Apr 4;14:45. doi: 10.1186/1471-2296-14-45 (PMC3662579; doi:10.1186/1471-2296-14-45)
Supplement: Additional file 2 — Price weights used for valuation of resource use, per visit unless otherwise mentioned. This file contains the price weights that were used to value resource use. [file 1471-2296-14-45-S2.pdf]

Additional file 2 - Price weights used for valuation of resource use, per visit unless otherwise mentioned

Type of utilization	Price weight[a]	Source of price weight
**Direct healthcare costs**		
Intervention: practice nurse		
Face to face session, 30 min<br>Phone session, 15 min	23.82<br>11.91	General practice collaborative labor agreement.
Primary care		
General practitioner	21.89[b]	Dutch costing manual[1]
Therapists	24.65 – 54.33[c]	Dutch costing manual[1]
Dietitian	14.50	Dutch costing manual[1]
Dentist	18.70	Dutch costing manual[1]
Primary mental health care	51.86 – 134.37[c]	Dutch costing manual[1]
Secondary care		
Outpatient visits	60.68	Dutch costing manual[1]
Admission general hospital (d)	365.18	Dutch costing manual[1]
**Direct patient costs**		
Complementary therapists	25.18 – 82.30[c]	Price according to professional organization
OTC	as reported by patient	-
Sports & sports equipment	as reported by patient	
**Indirect productivity losses**		
Sickness absence (d)	92 – 218[d]	Dutch costing manual[1]

[a] Euros, corrected to the year 2008, [b] Price for consultation at the practice; [c] Range of the price weights for the different providers, [d] Range of costs per sick leave (calendar) day, depending on age and sex

1. Oostenbrink JB, Bouwmans CAM, Koopmanschap MA, Rutten FFH: *[Dutch Manual for Costing: Methods and Standard Costs for Economic Evaluations in Health Care*]. Diemen, The Netherlands: Dutch Health Care Insurance Board; 2004.
