# Supplementary material for: Economic evaluation of a lifestyle intervention in primary care to prevent type 2 diabetes mellitus and cardiovascular diseases: a randomized controlled trial
Source: BMC Fam Pract. 2013 Apr 4;14:45. doi: 10.1186/1471-2296-14-45 (PMC3662579; doi:10.1186/1471-2296-14-45)
Supplement: Additional file 3 — Incremental cost-effectiveness ratios and distribution of the joint cost-effect pairs in the cost-effectiveness plane of all analyses. This file shows the results from the base case analysis and all sensitivity analyses. [file 1471-2296-14-45-S3.pdf]

Additional file 3 - Incremental cost-effectiveness ratios and distribution of the joint cost-effect pairs in the cost-effectiveness plane of all analyses

9-year risk for developing T2DM

Analysis[a]	Sample size per group		ΔC (95% CI)	ΔE (95% CI)	ICER	Distribution in CE plane (%)			
	Control	Inter-vention	Euros	T2DM risk		NE[b]	SE[c]	SW[d]	NW[e]
Base case	300	305	-866 (-2372;370)	0.6 (-0.1;1.3)	-1416	0.6	4.1	85.9	9.4
Human capital	300	305	-1048 (12868;448)	0.6 (-0.1;1.3)	-1714	0.6	4.2	85.6	9.7
Exclusion sports costs	300	305	-758 (-2258;442)	0.6 (-0.1;1.3)	-1239	0.7	3.9	82.8	12.6
Booster calls imputed	300	305	-846 (-2377;388)	0.6 (-0.1;1.3)	-1383	0.6	3.9	85.0	10.6
50 MI-sets	300	305	-873 (-2380; 397)	0.7 (-0.1;1.4)	-1301	0.5	3.5	86.1	9.9
Complete cases	117	105	-30 (-2171;1446)	0.7 (-0.4;1.7)	-44	5.1	4.4	45.0	45.5
Health care perspective	300	305	-5 (-316;272)	0.6 (-0.1;1.3)	-8	2.2	2.4	47.5	47.9

10-year risk for CVD mortality

Analysis[a]	Sample size per group		ΔC (95% CI)	ΔE (95% CI)	ICER	Distribution in CE plane (%)			
	Control	Inter-vention	Euros	CVD risk		NE[b]	SE[c]	SW[d]	NW[e]
Base case	300	305	-866 (-2372;370)	-0.1 (-0.4;0.2)	6405	8.0	74.3	15.4	2.3
Human capital	300	305	-1048 (12868;448)	-0.1 (-0.4;0.2)	7754	7.8	74.5	15.4	2.3
Exclusion sports costs	300	305	-758 (-2258;442)	-0.1 (-0.4;0.2)	5608	10.1	72.6	14.4	2.9
Booster calls	300	305	-846	-0.1	6260	8.5	74.1	15.0	2.3

Analysis[a]	Sample size per group		ΔC (95% CI)	ΔE (95% CI)	ICER	Distribution in CE plane (%)			
	Control	Inter-vention	Euros	CVD risk		NE[b]	SE[c]	SW[d]	NW[e]
imputed			(-2377;388)	(-0.4;0.2)					
50 MI sets	300	305	-873 (-2380; 397)	-0.1 (-0.4;0.2)	9915	7.2	66.0	23.6	3.2
Complete cases	116	104	-19 (-2253;1410)	-0.03 (-0.34;0.29)	642	29.5	27.8	21.3	21.5
Health care	300	305	-5 (-316;272)	-0.1 (-0.4;0.2)	38	40.1	42.4	8.0	9.5

QALYs gained

Analysis[a]	Sample size per group		ΔC (95% CI)	ΔE (95% CI)	ICER	Distribution in CE plane (%)			
	Control	Inter-vention	Euros	QALY		NE[b]	SE[c]	SW[d]	NW[e]
Base case	300	305	-866 (-2372;370)	0.02 (-0.02;0.05)	-50,273	8.2	76.8	12.9	2.1
Discounted	300	305	-851 (-2350;356)	0.02 (-0.02;0.05)	-49,397	8.3	77.2	12.4	2.2
Human capital	300	305	-1048 (12868;448)	0.02 (-0.02;0.05)	-60,856	8.2	77.0	12.5	2.3
Exclusion sports costs	300	305	-758 (-2258;442)	0.02 (-0.02;0.05)	-44,015	10.3	74.9	12.1	2.7
Booster calls imputed	300	305	-846 (-2377;388)	0.02 (-0.02;0.05)	-49,132	9.1	76.0	12.6	2.3
50 MI sets	300	305	-873 (-2380; 397)	0.02 (-0.01;0.05)	-54,335	7.5	78.5	11.1	3.0
Complete cases	114	98	110 (-2004;1611)	0.02 (-0.02;0.06)	4770	46.4	40.6	4.2	8.7
Health care perspective	300	305	-5 (-316;272)	0.02 (-0.02;0.05)	-298	40.7	44.7	5.0	9.6
Post hoc: sick leave truncated 30 days	300	305	-179 (-725;311)	0.02 (-0.02;0.05)	24,865	5.6	31.4	43.1	19.8

Explanation of abbreviations and footnotes

ΔC= mean difference in total costs between the intervention group and control group in Euros adjusted to the year 2009; ΔE= mean difference in outcome; ICER is calculated as ΔC/ ΔE. ICER, Incremental Cost-Effectiveness Ratio; NE, north-east; SE, south-east; SW, south-west; NW, north-west; T2DM, Type 2 Diabetes Mellitus; CVD, Cardiovascular Disease; QALY, Quality Adjusted Life Years.

[a] The main analysis and complete case analysis are based on the societal perspective. In the main analysis and the analysis from the health care perspective missing data were multiply imputed. The complete cases analysis was restricted to participants with complete data on costs and the particular clinical outcome. [b] NE quadrant: the intervention is more effective and more costly than usual care. [c] SE quadrant: the intervention is more effective and less costly than usual care. [d] SW quadrant: the intervention is less effective and less costly than usual care. [e] NW quadrant: the intervention is less effective and more costly than usual care.
